# Asylum Matters

**Published:** 1900-11-24

**Authors:** 


### ASYLUM MATTERS.

A correspondent, writing on another aspect of this case, says:—

"However suitable the wards of workhouse infirmaries are, or might be, for harmless imbeciles, it is unquestionable that they are ill adapted for the treatment, temporary or permanent, of acute cases of insanity, and such patients ought not to be kept in them. This truism has been again demonstrated by the trial of attendant George Pescott, of the Crumpsall Workhouse. The evidence went to show that Pescott had fastened a towel round the neck of a patient, and had used a poker to twist the towel tightly. The patient seems to have been a noisy and refractory general paralytic, in which disease collapse is sometimes easily induced, and death took place. The jury returned a verdict of manslaughter, and Pescott was sentenced to penal servitude for seven years.

"Mr. Byrne, the barrister for the defence, very pertinently asked why 120 certified lunatics were kept in the workhouse; and he received the old stereotyped answer, that there was no room in the County Asylum. Now, if there is one thing more certain than another about insanity in any particular county, it is that the rate of increase of the certified insane can be predicted with almost unerring accuracy; and we should like to ask the County Council of Lancashire why the required accommodation was not provided. It must be well-nigh ten years since the Borough of Blackburn and the County of Lancashire both decided to build asylums, and yet we have not learnt that either of these was completed. The latter is understood to be a very large one, and had it been opened earlier, or another one built if it be already opened, it is conceivable that the trial and sentence of Pescott might never have been necessary, as the patient would have been in the County Asylum. We should further like to ask what steps are taken by the Crumpsall Workhouse authorities to train their attendants. A very small amount of knowledge is sufficient to convince any nurse that a general paralytic must be tenderly handled, and that humane treatment of the insane is alone permissible in any case."

